# Development of a diagnostic and risk prediction model for Alzheimer’s disease through integration of single-cell and bulk transcriptomic analysis of glutamine metabolism

**DOI:** 10.3389/fnagi.2023.1275793

**Published:** 2023-11-10

**Authors:** Yan Guo, Tingru Zhao, Xi Chu, Zhenyun Cheng

**Affiliations:** ^1^Department of Clinical Laboratory, Key Clinical Laboratory of Henan Province, Zhengzhou, Henan, China; ^2^The First Affiliated Hospital of Zhengzhou University, Zhengzhou, Henan, China

**Keywords:** single-cell, glutamine metabolism, Alzheimer’s disease, machine learning, immune response

## Abstract

**Background:**

In this study, we present a novel system for quantifying glutamine metabolism (GM) to enhance the effectiveness of Alzheimer’s disease (AD) diagnosis and risk prediction.

**Methods:**

Single-cell RNA sequencing (scRNA-seq) analysis was utilized to comprehensively assess the expression patterns of GM. The WGCNA algorithm was applied to investigate the most significant genes related to GM. Subsequently, three machine learning algorithms (Boruta, LASSO, and SVM-RFE) were employed to identify GM-associated characteristic genes and develop a risk model. Patients were divided into high- and low-risk groups based on this model. Moreover, we explored biological properties, distinct signaling pathways, and immunological characteristics of AD patients at different risk levels. Finally, *in vitro* and *in vivo* models of AD were constructed to validate the characteristics of the feature genes.

**Results:**

Both scRNA-seq and bulk transcriptomic analyses revealed increased GM activity in AD patients, specifically in certain cell subsets (pDC, Tem/Effector helper T cells (LTB), and plasma cells). Cells with higher GM scores demonstrated more significant numbers and strengths of interactions with other cell types. The WGCNA algorithm identified 360 genes related to GM, and a risk score was constructed based on nine characteristic genes (ATP13A4, PIK3C2A, CD164, PHF1, CES2, PDGFB, LCOR, TMEM30A, and PLXNA1) identified through multiple machine learning algorithms displayed reliable diagnostic efficacy for AD onset. Nomograms, calibration curves, and decision curve analysis (DCA) based on these characteristic genes provided significant clinical benefits for AD patients. High-risk AD patients exhibited higher levels of immune-related functions and pathways, increased immune cell infiltration, and elevated expressions of immune modulators. RT-qPCR analysis revealed that the majority of the nine characteristic genes were differentially expressed in AD-induced rat neurons. Knocking down PHF1 could protect against neurite loss and alleviate cell injury in AD neurons. *In vivo*, down-regulation of PHF1 in AD models decreases GM metabolism levels and modulates the immunoinflammatory response in the brain.

**Conclusion:**

This comprehensive identification of gene expression patterns contributes to a deeper understanding of the underlying pathological mechanisms driving AD pathogenesis. Furthermore, the risk model based on the nine-gene signature offers a promising theoretical foundation for developing individualized treatments for AD patients.

## Introduction

AD is a neurodegenerative syndrome that worsens progressively with age. The most common clinical manifestation of AD is a slowly progressing amnestic disorder, which reflects a neurofibrillary tangle pathology. This pathology is mainly distributed early in the medial temporal lobe structures and eventually develops into a multidomain dementia, with amnesia being the predominant feature (Scheltens et al., 2021). Neurodegenerative dementia is predominantly caused by AD, accounting for 50–70 percent of all cases. Unfortunately, more than 50 million people worldwide are currently suffering from neurodegenerative disorders, and this number is expected to triple by 2050 if no effective preventative or therapeutic solutions are found (Winblad et al., 2016). Although various treatment strategies have been explored in clinical trials for decades, the significant heterogeneity of AD has led to the availability of primarily symptom-based treatments rather than actual curative therapies (Author, 2021; Passeri et al., 2022). AD is known to start at least 20 years before symptoms appear. Moderate cognitive impairment (MCI) is a common stage of the progression of AD. However, not all patients with MCI develop AD, suggesting that there may be protective or causative variables that affect different groups of patients, even within conventional categories (Braak et al., 2011; Lombardi et al., 2020). Moreover, research suggests that over 30% of AD cases globally may be linked to modifiable risk factors, both genetic and acquired (Scheltens et al., 2021). These factors could serve as targets for prevention interventions aimed at reducing the risk of cognitive decline associated with AD (Silva et al., 2019; Lombardi et al., 2020). Therefore, it is essential to understand the heterogeneity of AD and accurately distinguish the molecular characteristics of each patient to guide personalized treatment.

Recent longitudinal studies have partially attributed the decline in the incidence of dementia to improved control of metabolic factors (Satizabal et al., 2016; Rejc et al., 2022). Glutamine, which is the most abundant and versatile amino acid in the human body, is often used to maintain TCA flux in cellular aerobic glycolysis or as a source of citric acid for lipid synthesis in reductive carboxylation (Cruzat et al., 2018). The glutamate-glutamine cycle is recognized as a crucial element for the transmission of excitatory singles in the central nervous system (CNS), impacting cognition and other brain processes, as well as the neurodegenerative process (Sidoryk-Wegrzynowicz and Aschner, 2013). Insufficient levels of glutamine and glutamate in the brain have been proposed as possible biomarkers for AD (González-Domínguez et al., 2015; Stevenson-Hoare et al., 2023). A two-sample Mendelian randomization study indicated that circulating glutamine can act as a neuroprotectant and that modifications in glutamine/glutamate metabolism may prevent cognitive decline in Alzheimer’s disease (AD) (Sun et al., 2022). Animal models of AD have shown that disruption of glutamine homeostasis is related to the severity of cognitive impairment (Baek et al., 2023; Sun et al., 2023). However, two cohort studies found that a higher serum glutamine level was significantly associated with an increased risk of AD (van der Lee et al., 2018; Cui et al., 2020). These studies suggest that glutamine metabolism may play a complicated role in the onset and progression of AD. However, the landscape of glutamine metabolism in AD is still largely unknown. Targeting GM alongside AD treatment has shown promising results (Adams, 2020; Dejakaisaya et al., 2021; Sun et al., 2022). Therefore, we conducted this study to systematically analyze glutamine metabolism in AD.

Our research comprehensively evaluated the expression patterns of GM-related variants in AD patients. A single-cell approach was applied to display the GM landscape of various cell types and intracellular communication in AD samples with different levels of Gln. bulk-RNA-Seq-based weighted gene co-expression network analysis (WGCNA) was performed to identify GM-associated gene modules, and then three machine learning methods, including Boruta, Least Absolute Shrinkage and Selection Operator (LASSO), and SVM Recursive Feature Elimination (SVM-RFE), were employed to explore characteristic genes. Then a GM-related risk scoring system and a nomogram scoring system were constructed, and biological traits, involved pathways, and immunological features were explored in AD patients at different risk levels. We also verified the expression of nine signature genes by RT-qPCR and the Geo database. Finally, we used lentivirus-mediated gene knockdown technology to conduct further functional studies on the most representative feature gene in an *in vitro* model of AD.

Altogether, our study highlighted significantly the relationship between GM expression patterns and AD heterogeneity, providing unique insights into how to treat AD patients on an individualized level.

## Materials

### Data acquisition and preprocessing

The peripheral blood scRNA-seq data GSE181279 (based on the GPL24676 platform), including three AD patients and two age-matched cognitive normal controls (NC), was obtained from the Gene Expression Omnibus (GEO) database (Xu and Jia, 2021). The “NormalizeData” function in the R Seurat package was used to normalize the expression matrix. Then integrated analysis and elimination of batch variation were performed using the harmony R package. Afterwards, principal component analysis (PCA) and uniform manifold approximation and projection for dimensionality reduction (UMAP) were adopted for dimensionality reduction of the combined data. High-quality scRNA-seq data was filtered using the following parameters: Genes were expressed in more than three cells per cell, with 200 to 5,000 genes and not more than 15% mitochondrial genes. After filtering, a total of 34,738 cells were selected for the following analysis. The top 4,000 most variable genes were then identified using the vast technique included in the Seurat package’s “Find Variable Features” function. The “Find Clusters” tool was used to cluster the cells after embedding them in the PCA space’s graph structure, and cell annotations were predicted using the CellTypist Python package. Furthermore, the differentially expressed genes (DEGs) of each cluster were determined using the “Find ALL Markers” function of the “Seurat” program with logfc. Threshold = 0.5. The bulk transcriptome data (GSE33000 and GSE5281) were also acquired from the GEO. In brief, GSE33000 is based on the GPL4372 platform and consists of 157 normal and 319 AD brain tissue samples, while GSE5281 is built on the GPL570 platform and contains 74 normal and 87 AD brain tissues (Blalock et al., 2011; Narayanan et al., 2014). The raw data from each dataset were then normalized and log-transformed using the robust multi-array averaging (RMA) function of the “affy” R package.

### Construction of a GM score

According to previous studies, we extracted 118 regulators as the biomarkers of GM (Liberzon et al., 2015; He et al., 2022). Additionally, the single-cell-based and bulk RNA-based GM scores were calculated using the “Ucell” and single-sample gene set enrichment analysis (ssGSEA) algorithms, respectively. Differences in the distribution of high and low glutamine metabolism at the single-cell level in the AD group were bound by the 75% quantile of GM scores, and the result was illustrated using UMAP plots.

### Cell-cell interaction analysis

The “CellChat” R package was utilized to investigate cell–cell communications and essential pathways with default parameters based on the UMI count matrix for each group (High_Gln and Low_Gln). “CellChatDB.human” was employed as the database of receptor-ligand interactions. The “mergeCellChat” function was applied to combine CellChat objects from each group to evaluate the total number of interactions and the strength of interactions. Then the “netVisual DiffInteraction” function was used to visualize the variations between groups in the quantity or intensity of cell–cell interactions. Finally, the “rankNet” function was chosen to identify signaling pathways with differential expression, and the “netVisual bubble” and “netVisual aggregate” functions were applied to display the distribution of gene expression between groups.

### The weighted gene co-expression network analysis

The weighted gene co-expression network analysis (WGCNA) method was used to investigate the gene expression modules associated with the GM score through the “WGCNA” R package. The brief processes were as follows: Gene expression landscapes from GSE33000 were chosen as input data, and the ideal soft threshold for adjacency computation was then immediately identified. Next, to determine the genetic connectivity of the network, the expression matrix was transformed into an adjacency matrix and a topological overlap matrix (TOM). Then, based on the TOM values, the genes in the matrix were hierarchically clustered to generate clustering trees. Modules with strong associations with the GM score were ultimately evaluated and chosen for further analysis.

### Enrichment analysis

Enrichment analysis, including Gene Ontology (GO), biological process (BP), cellular components (CC), and molecular functions (MF), and the Kyoto Encyclopedia of Genes and Genomes (KEGG), was performed via the ClusterProfiler R package. For the enrichment results, the value of *p* was corrected based on the Benjamini-Hochberg method, and an adjusted *p* < 0.05 was considered significant.

The “GSVA” R package was utilized to conduct Gene Set Variation Analysis (GSVA) enrichment to assess the heterogeneity of a variety of biological processes and pathway activities. The MSigDB hallmark gene sets “c2.cp. Kegg.v7.4.symbols” and “c5.go.bp.v7.5.1.symbols” were selected as the preferred gene sets for GSVA. The “limma” R package was used to calculate the variances in biological functions and signaling pathways, and absolute t-values for GSEA scores >2 were considered to be statistically significant.

Furthermore, we performed gene set enrichment analysis (GSEA) to evaluate the differences in pathway activity depending on the “GSEA” R package. Normalized enrichment scores (NES) were sorted, and adjusted *p* values <0.05 were deemed significant.

### Screening of characteristic GM-related genes based on machine learning

Three machine learning algorithms, namely Boruta, SVM Recursive Feature Elimination (SVM-RFE), and Least Absolute Shrinkage and Selection Operator (LASSO) were utilized to identify potential features associated with GM. The R packages ‘Boruta’, “e1071” and ‘caret’ were used for this purpose. The Boruta algorithm was used for identifying important GM-related gene signatures, with parameters set at 300 iterations and a value of *p* less than 0.01. The identified gene signatures were then further analyzed using LASSO and SVM-RFE algorithms. The LASSO model was used to determine the final coefficients of important variables, based on the optimal penalty parameter γ. To avoid over-fitting, SVM-RFE and LASSO were conducted with default parameters using 10-fold cross-validation. The AD brain tissue samples in the GSE33000 dataset were randomly divided into a training cohort (70%) and a verification cohort (30%). The final GM-related characteristic genes were determined by intersecting the results of LASSO and SVM-RFE. To evaluate the effectiveness of different machine learning methods, we utilized the ‘pROC’ R package to calculate the area under the receiver operating characteristic curve (AUC) for both the GSE33000 and GSE5281 datasets.

### Nomogram and risk model establishment based on GM-related characteristic genes

A predictive nomogram was created using the R package ‘rms’, which included riskScore and clinical factors such as age and gender. To evaluate the precision of the nomogram, a calibration curve was drawn. The clinical utility of the nomogram was quantified and evaluated using the R package ‘ggDCA’, which produced the results of the decision curve analysis (DCA).

GM-related riskScore were determined using a LASSO machine learning model. The formula used to calculate the risk score was: riskScore = ∑_i_Coefficients_i_ × Expression level of characteristic genes_i_. Based on the median value of the risk score, AD patients were divided into two groups: high-risk and low-risk.

### Immune microenvironment evaluation

The ssGSEA algorithm included in the R “GSVA” package was employed to estimate the immune infiltration levels and quantify the fractional enrichment or relative abundance of 28 immune cell subtypes in different risk groups. The Wilcoxon rank-sum test was used to compare the levels of immune scores between groups. In addition, immune scores for each group were calculated via the R package “ESTIMATE.” The difference in the expression levels of 60 immunoregulatory genes (antigen presentation, cell adhesion, co-inhibitor, co-stimulator, ligand, and receptor) and between groups was further displayed using a heat map. The correlation between 28 immune cell subtypes and the riskScore was elucidated by Pearson correlation analysis. This study was approved by the Institutional Animal Care and Use Committee of Zhengzhou University and conducted following the Guidelines for the Care and Use of Laboratory Animals.

### Construction of a cellular model of AD in primary cortical neurons

Primary cortical neurons were cultured as described previously (Chen et al., 2019). Briefly, cerebral cortex samples were isolated from the brains of Sprague–Dawley rat embryos (16–18 days). Cell suspensions were plated on 6-well plates with poly-L-lysine and cultured in neurobasal medium (Gibco, USA) containing 2% B27 (Gibco, USA), 0.5 mM glutamine, and 50 U/mL penicillin/streptomycin, and neurons were cultured at 37°C and 5% CO2. The neurobasal medium was first refreshed after 12 h, and then half of the medium was refreshed every two days. In a 37°C, 5% CO2 incubator, neurons were cultured for 7–9 days.

Oligomeric Aβ_1-42_ was prepared according to previous research with minor modifications (Stine et al., 2011; Jayatunga et al., 2021). First, Aβ_1-42_ was dissolved in pre-cooled hexafluoroisopropanol (HFIP) at a concentration of 1 mM. The Aβ_1-42_-HFIP solution was incubated at room temperature for 30 min, followed by 10 min on ice. Then, the solution was dispensed into microcentrifuge tubes and placed in a fume hood to air dry overnight. The Aβ_1-42_ peptide films in tubes were dissolved in DMSO (Gibco, USA) and stored at −20°C before use. Next, pre-cooled F-12 (Gibco, USA) medium was added to each tube to maintain oligomerization conditions, and they were incubated at 4°C overnight for 24 h. After centrifugation and removal of the precipitate, the supernatant containing Aβ_1-42_ peptide was prepared for subsequent experiments. Finally, neurons were incubated with 20 umol/L Aβ_1-42_ oligomer at 37°C for 12 h, then cultured in a replaced neurobasal medium before harvest.

### Lentivirus transfection

Before Aβ1-42 oligomer exposure, neurons in culture were transfected with rat PHF1 shRNA lentivirus or a non-targeting scramble shRNA lentivirus (Genechem, Shanghai, China) at an MOI of 5, as per the manufacturer’s guidelines. The PHF1 shRNA target sequences were as follows: 5′ -GATCAUUGAUTTTTTGGAAC-3′. Scrambled shRNA was used as the control, with sequences: 5′-TGTGATGTCTCTCAT-3′. Post-transfection, neurons were processed for various experiments after 48 h. RT-qPCR was applied to confirm the knockdown efficiency. The experiment groups were named as Control, AD model (AD), AD + normal control scramble shRNA lentivirus (AD+ le-shNC), AD + PHF1 shRNA lentivirus (AD+Le-shRNA). All the following experiments *in vitro* were repeated at least three times.

### Animals

All experiments involving animals were performed following approved guidelines and ethical approval from the Animal Care and Use Committee and according to the NIH Guidelines for the Care and Use of Laboratory Animals (NIH publication, 1996). Two-month-old Sprague Dawley (SD) male rats were housed three-per-cage in an animal lab under standard conditions (12-h light/dark cycle in a room at a temperature of 20–25°C) with access to food and water adlibitum.

### Construction of an *in vivo* model of AD

To induce Alzheimer’s disease in the present study, beta-amyloid 1–42 (Aβ1-42) was injected into the CA1 region of the dorsal hippocampus of Aβ rats using a stereotaxic device (RWD, China). To prepare the injection solution, 1 mg/mL of Aβ1-42 (Abcam, Cambridge, UK, Cat no. ab120301) was initially diluted with ice-cold 1,1,1,3,3,3-hexafuoro-2-propanol (HFIP) and incubated at room temperature for at least 60 min with occasional vortexing. Next, the HFIP was removed using a SpeedVac, and the peptide flm was stored at−80°C. Ten for aggregation, the peptide was first resuspended in DMSO (5 mM), sterile phosphate buffer saline (PBS) was added to bring the peptide to a final concentration of 1 μg/μL, and the Aβ1-42 peptide was incubated at 37°C for 72 h. To perform stereotaxic surgery, the animals were anesthetized with isofurane inhalation. Aβ1-42 suspension (1 μL/site) was injected into the CA1 region of the dorsal hippocampus of A-C, and A-Ex groups (A-4.2, L ± 3.0, V-2.0 mm) based on the Paxinos and Watson atlas by a Hamilton syringe attached to an infusion pump12. Moreover, isotonic saline solution was injected into the control animals.

Four weeks after the AβO injection, a PHF1 knockdown model was developed in AD rats to endorse the role of PHF1 in Alzheimer’s disease (AD). In this process, adenoviral vectors containing short hairpin RNA (shRNA) targeting the rat PHF1 gene (Ad-shPHF1), and scrambled negative control shRNA (Ad-shNC) were acquired from RiboBio (Guangzhou, China). For individual rats, about 3×10^10 plaque-forming units (PFU) of Ad-shPHF1, dissolved in 200 μL of normal saline, were administered via tail vein injection. This substance served as the treatment group’s remedy, while an equal quantity of Ad-shNC was assigned to the control group. On the 14th day post-injection, the efficiency of the knockout was assessed through quantitative real-time PCR (qRT-PCR).

### Collection of brain samples

The rats were anesthetized with carbon dioxide and then decapitated. The brain was subsequently removed and washed with distilled water. Using an electric homogenizer, the whole brain was homogenized in bidistilled water.

### Enzyme-linked immunosorbent assay (ELISA)

Rat brain glutamine (Gln), and inflammatory cytokines (IL-6, IL-10, IFN-γ and TNF-α) were measured by using ELISA kits from Cusabio (Wuhan, China).

### Real-time RT-PCR analysis

Total RNA was extracted from cultured primary cortical neurons and rat brains using Trizol reagent (Life Technologies, USA), and then reverse-transcribed into complementary DNA using Revert Aid First Strand cDNA Synthesis Kit following the manufacturer’s instructions. The quantitative RT-PCR was performed with the ABI PRISM 7500 real-time PCR system (Applied Biosystems, USA) using the SYBR Premix EX Taq (Takara, Japan). Primer sets were displayed as follows:

ATP13A4: 5′ -GTCAAGCCTGGTGGAAGAAT-3′ (forward) and 5’-CTGATAACGAGACGCCAACAT-3′ (reverse);
PIK3C2A: 5’-GGAGGAGCAGTGAAGTTATCG-3′ (forward) and 5’-GGTATCTGGAAGCAGGTATGTT-3′ (reverse);
CD164: 5’-GCATCTCCCAACGTGACTGA-3′ (forward) and 5’-GGCGTTGACACAGGAAACAC-3′(reverse);
PHF1: 5’-CCATCTTGTTCTCTACCATCTCAG-3′ (forward) and 5’-CGGTCCTTGTGGCTGTTAAG-3′(reverse);
CSE2: 5’-TTGACTTCACTGAGGAGGAGAG-3′ (forward) and 5’-CTGCTCATCGTGGTCCAATG-3′(reverse);
PDGFB: 5’-CTGCTACCTGCGTCTGGTC-3′ (forward) and 5’-TGCTCGGGTCATGTTCAAGT-3′(reverse);
LCOR: 5’-CTGCCTTCCTCAACCTACAAC-3′ (forward) and 5’-GTGTCTGAATCTTCCTCGTCTAAG-3′(reverse);
TMEM30A: 5’-TGCGTACTGCAGCTTTACCT-3′ (forward) and 5’-TGGATTCTTTCCTCCCATCCA-3′(reverse);
PLXNA1: 5’-TCGAATCCGGGCCAAGTATG-3′ (forward) and 5’-CAATCTCATCTGGCCGCTCT-3′(reverse).

The cycle threshold (CT) of the target gene was normalized to the CT value of β-actin, and the results were expressed as fold changes using the 2^−ΔΔCT^ method to evaluate the relative quantification of mRNA expression.

### Immunofluorescence staining

To ascertain the role of PHF1 in neurite outgrowth in AD, we performed immunofluorescence staining to observe neurite outgrowth, as previously described (IL-10). At day 15, neurons were rinsed with PBS three times and fixed with 4% paraformaldehyde (pH 7.4) for 15 min. Cells were then subjected to overnight incubation at 4°C with the primary antibody: mouse anti-class III-β-Tubulin antibody (1400, Beyotime, China). Subsequently, the cells were washed three times with PBS and incubated with corresponding secondary antibodies (Dylight488 donkey anti-mouse IgG, 1:800, Jackson Immunoresearch, USA) for 2 h at room temperature. Nuclei were stained with Hoechst33342 (5 μg/mL, Sigma, USA). The slides were examined under a ZEISS LSM 780 confocal microscope (Carl Zeiss, Germany), and quantification was performed using the LSM Image Browser (V4.2.0.121) to assess the number and length of primary neurites originating directly from the soma.

### Cell viability assay

To assess the degree of cell injury after PHF1 knockdown in the AD model. A Cell Counting Kit from Seven Sea Biotech, Shanghai, China was used to assess cell viability, with strict adherence to the manufacturer’s instructions. Briefly, 50 μL of CCK-8 solution was added to each 500 μL of medium in a 24-well plate and was incubated for 4 h at a temperature of 37°C. Absorbance was then measured at 450 nm using an Infinite M200 microplate reader (TECAN).

### LDH release assay

The degree of cell injury in each group was also assessed using the LDH-Cytotoxicity Colorimetric Assay Kit II (#K313-500, BioVision, USA), according to the manufacturer’s instructions. Briefly, 10 μL of medium per well was added to an optically clear 96-well plate. 100 μL of LDH Reaction Mix was then added to each well, mixed well, and left to react at room temperature for 30 min. Absorbance measurements were made at 450 nm using an Infinite M200 microplate reader (TECAN).

### Statistical analysis

The statistical analysis of this study was performed via R 4.1.0 software. Differences between groups were compared by Wilcoxon, sum-rank test, or *t*-test. The correlation between two continuous variables was elucidated by Pearson correlation analysis. The ROC curve analysis was used to predict binary categorical variables. The value of *p* was adjusted for the false discovery rate (FDR) using the Benjamin-Hochberg method, and two-sided *p* < 0.05 were considered statistically significant.

## Results

### Analysis of GM at the level of single-cell

**Figure 1:**
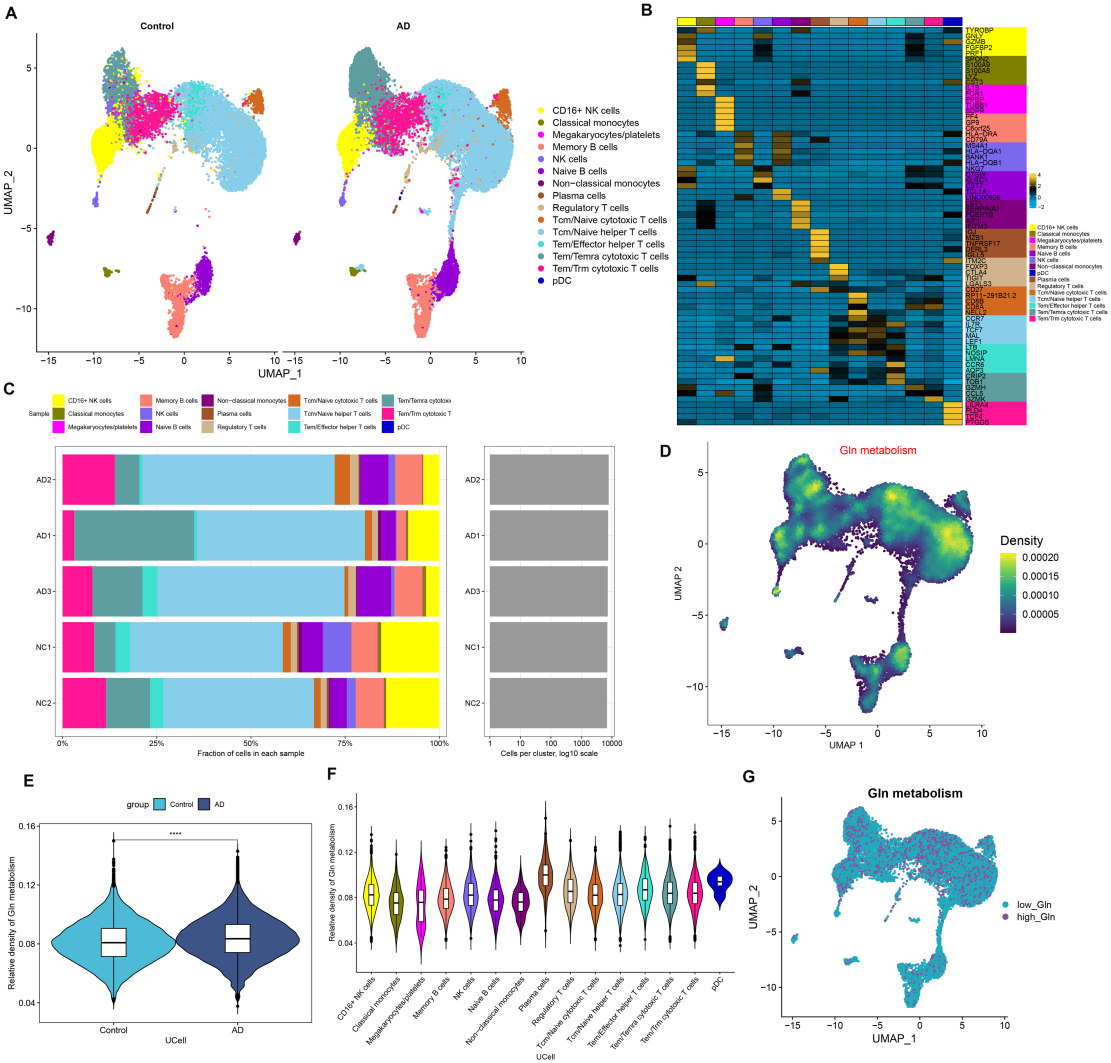
Characteristic of GM at the single-cell level. **(A)** The UMAP plot of GSE181279 illustrated the distribution of 15 major cell clusters in each group. **(B)** The top five differential expressed genes per cell type were visualized with a heat map. **(C)** Cell type fraction of each sample in GSE181279. **(D)** Differences in GM score based on the UCell algorithm were presented via UMAP plots. **(E)** The violin diagram demonstrated the differences in GM scores between the AD and control groups. **(F)** The violin diagram exhibited the differences in GM score among each cell type. **(G)** UMAP plot demonstrated the difference in the distribution of high and low GM at the single-cell level in the AD group, bounded by the 75% quantile of the GM score.

We first investigated the differences in glutamine metabolic activity of different cell types. A total of 34,738 filtered cells were grouped into 12 cell clusters, including CD16+ NK cells, classical monocytes, megakaryocytes/platelets, memory B cells, NK cells, Naive B cells, non-classical monocytes, plasma cells, regulatory T cells, Tcm/Naive cytotoxic T cells, Tcm/Naive helper T cells, Tem/Temra cytotoxic T cells, Tem/Trm cytotoxic T cells, and PDC (Figure 1A). The top 5 DEGs for each cell cluster were exhibited by a heat map (Figure 1B). Cell type fractions of each sample are shown in Figure 1C. The “UCell” algorithm was then applied to calculate the GM score for each cell subset, and the results demonstrated that AD samples had a higher GM score in particular cell subsets, particularly in the plasma cell, Tem/Effector helper T cells (LTB), and pDC (plasmacytoid dendritic cells) regions (Figures 1D–F). Subsequently, filtered cells in the AD group were divided into the high-glutamine group and the low-glutamine group based on the 75% quantile of the GM score (Figure 1G).

### Intercellular communication analysis

**Figure 2:**
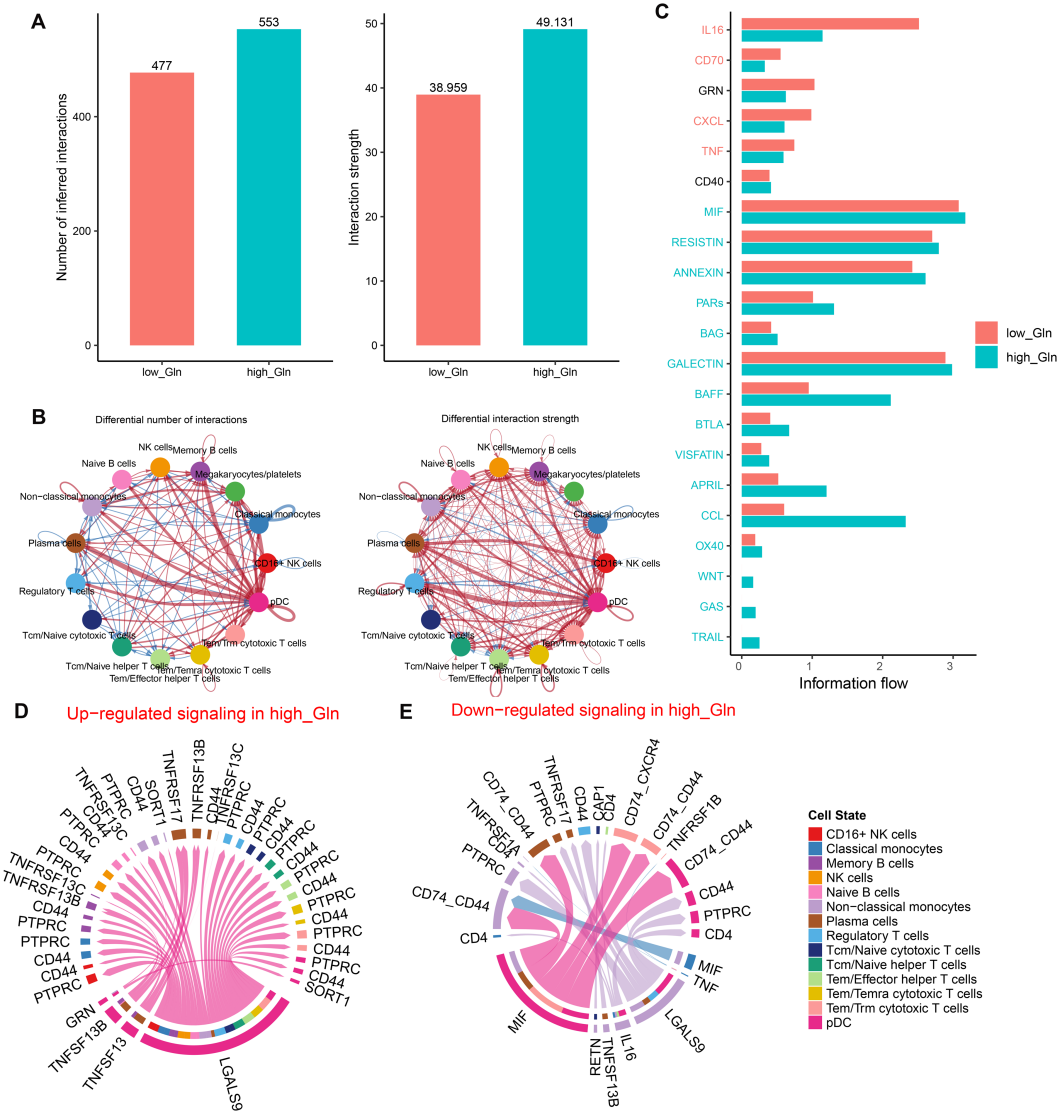
Intercellular communication differences between the high-glutamine and low-glutamine groups. **(A,B)** Charts with bars and circles depicted the variations between low-glutamine and high-glutamine groups in the number of interactions (left) or strength of interactions (right) in the network of cell–cell communication. Stronger interactions were represented by thicker lines and increased or decreased singling in the high-glutamine group when compared to the low-glutamine group was represented by red or blue colors, respectively. **(C)** Stack plots depicted the variations in intercellular singling networks between the high- and low-glutamine groups. Orange and green colors denote up-regulated signaling pathways in low- and high-glutamine groups, respectively. The X-axis represented the relative enrichment degree of the signaling pathway. **(D,E)** Chord plots indicated the difference in singling molecules between each cell cluster in the high-glutamine group.

To determine possible interactions between cells in low-glutamine and high-glutamine groups, CellChat analysis was performed. Larger interaction numbers and greater interaction strength were represented in the high-glutamine group (Figure 2A). Compared to the low-glutamine group, memory B cells, megakaryocytes/platelets, plasma cells, and pDC cells in the high-glutamine group showed greater interaction counts and interaction strengths than other cell types. In the low-glutamine group, monocytes and pDC cells predominantly communicated with classical monocytes and regulatory T cells (Figure 2B). Next, the specific signaling pathways were further identified between the two groups. In the high-glutamine group, signaling pathways including MIF, RESISTIN, ANNEXIN, PARs, BAG, GALECTIN, BAFF, BTLA, VISFATIN, APRIL, CCL, OX40, WNT, GAS, and TRAIL were more active compared with the other group. Among them, BAFF, CCL, and APRIL exhibited particularly high activity. Interestingly, WNT, GAS, and TRAIL were active only in the high glutamine group. In addition, IL-16, CD70, CXCL, and TNF signal pathways were significantly more active in the low-glutamine group (Figure 2C). Furthermore, we explored communication probabilities between cells in the high-glutamine group. We found that pDC and non-classical monocytes had great communication probabilities with other cells. In brief, except for plasma cells, pDC interacted with the majority of cells by up-regulating LGALS9-PTPRC/CD44/SORT1 signaling pathways, while pDC interacted with plasma cells and memory cells by binding TNFRSF13 and up-regulating TNFRSF13B/TNFRSF13C/TNFRSF13 (Figure 2D). pDC interacted with non-classical monocytes, plasma cells, Tem/Trm cytotoxic T cells, and pDC itself by down-regulating MIF-CD74-CXCR4/CD74-CD44. Non-classical monocytes interacted with other cells by down-regulating LGALS9-CD4/CD44/PTPRC, IL-16-CD4, TNFRSF13B-TNFRSF17, and RETN-CAP1 (Figure 2E).

### Investigation of GM-associated genes

**Figure 3:**
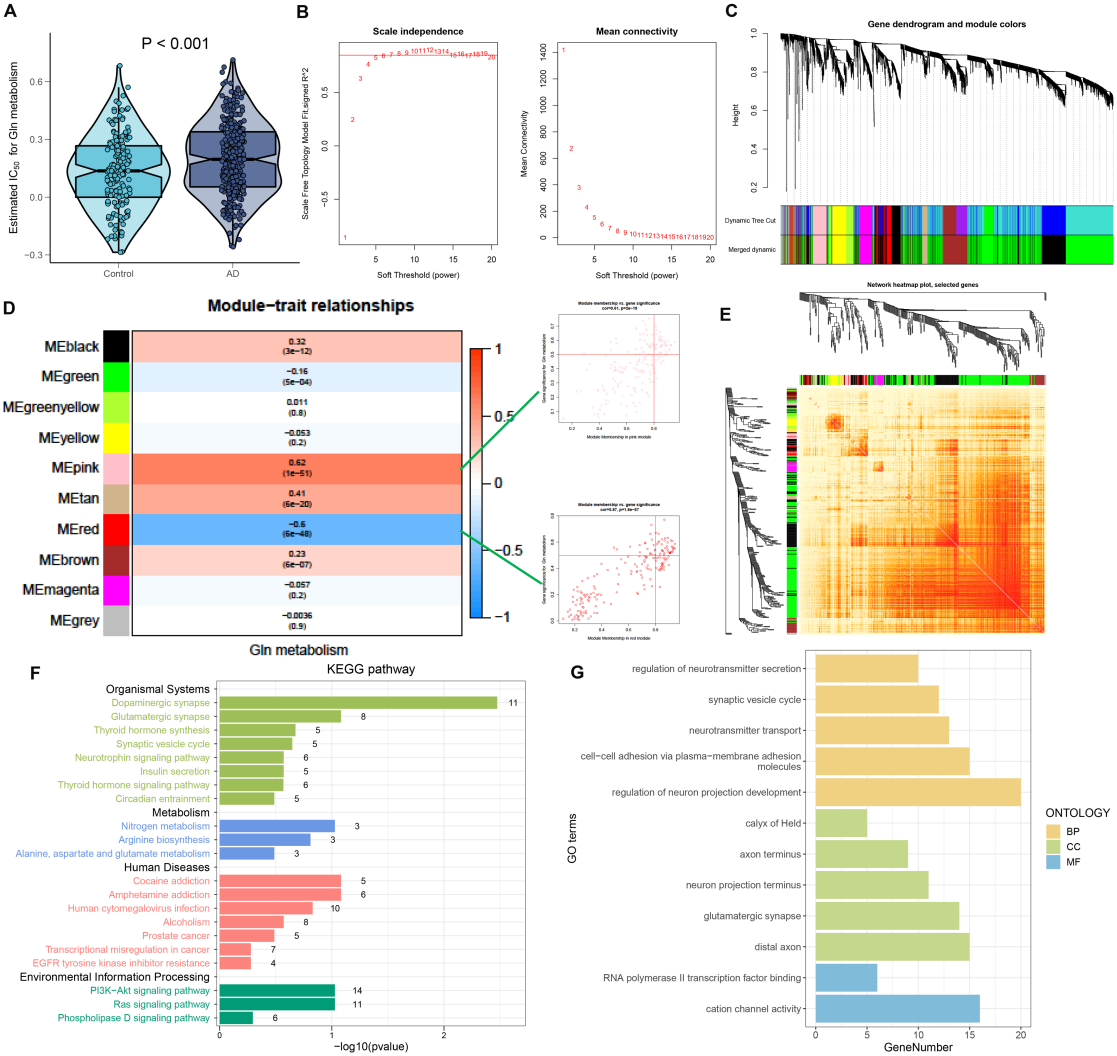
Identification of GM-associated genes. **(A)** GM score established via the ssGSEA algorithm to compare differences in GM levels between the Control and AD groups in the GSE33000 dataset. **(B)** The selection of soft threshold power. **(C)** Dendrogram of co-expression module clustering. **(D)** The WGCNA analysis investigated the modules that were most closely related to the GM score. Scatter plots represented the module membership (pink or red) and gene significance of GM. **(E)** A representative heatmap of the correlations among 10 modules. **(F)** KEGG analysis of 360 genes from the pink and red modules. **(G)** GO analysis of 360 genes from the pink and red modules.

To further investigate the relationship between GM and AD, we calculated the GM score for each simple in GSE 33000 utilizing the ssGSEA algorithm. Interestingly, we discovered that samples from the AD group had greater GM scores than those from the control group, which consisted of single-cell analysis results (Figure 3A). Subsequently, we performed the WGCNA algorithm to further determine the regulatory pattern of GM and verified GM-related genes based on their expression profiles in GSE33000. In detail, a total of 10 modules containing 4,425 genes were finally identified, and the heatmap displayed the correlation between each module and the GM score (Figures 3B–E). Modules with high correlation coefficients (177 genes in the pink module and 183 genes in the red module) were selected for subsequent analysis (Figure 3D). KEGG and GO analysis demonstrated that the 360 module-related genes were broadly involved in some human diseases, signaling pathways related to environmental information processing, organismal systems, and some metabolic pathways (Figure 3F). Moreover, the most significant enrichment functions were neurotransmitter secretion, cell–cell adhesion, neurotransmitter transport, neuron projection development (a biological process), calyx of Held, axon terminus, neuron projection terminus, glutamatergic synapse, distal axon (a cellular component), RNA polymerase ll transcription factor binding, and cation channel activity (a molecular function) (Figure 3G).

### Selection of characteristic GM-associated genes via machine learning approaches

**Figure 4:**
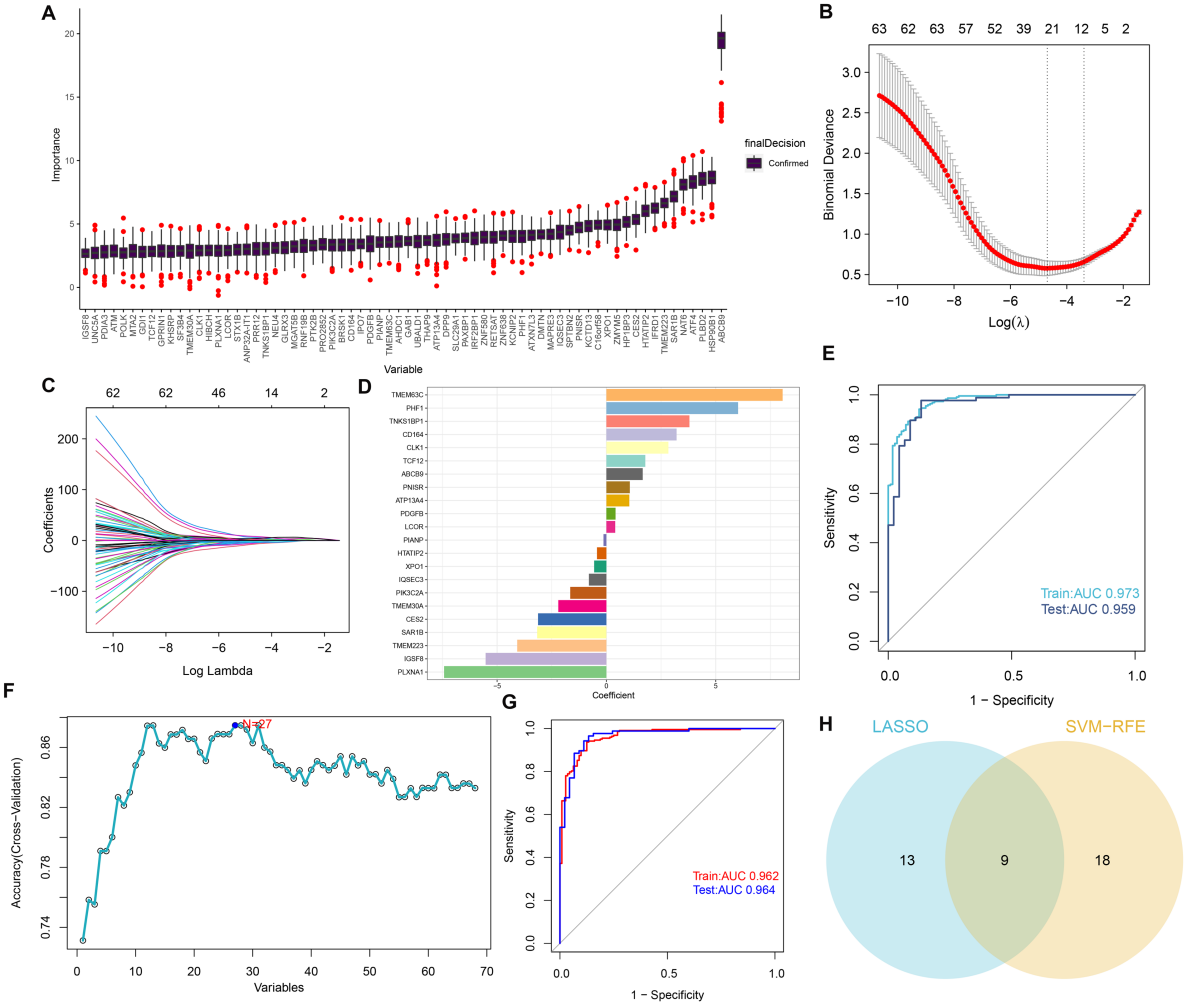
Selection of features associated with GM. **(A)** The Boruta analysis validated 68 important variables as candidates. **(B)** Tuning feature selection in the LASSO model. The vertical coordinate is the value of the coefficients, the subscript is log (lambda), and the superscript is the number of non-zero coefficients in the model at this point. **(C)** LASSO coefficient profiles of the feature genes associated with GM. **(D)** The specific coefficient value of the 22 genes associated with GM, is identified by the optimal lambda value. **(E)** The ROC curves and AUC values of the 22-gene-based model constructed by the LASSO algorithm in the training and testing cohorts. **(F)** SVM-RFE algorithm in the training and testing cohorts. **(G)** The ROC curves and AUC values of the 27-gene-based model constructed by the SVM-RFE algorithm in the training and testing cohorts. **(H)** Venn plot of feature genes selected by LASSO and SVM-RFE.

To determine potential markers for the diagnosis of AD, we employed three machine learning algorithms to identify characteristic features associated with GM. First, based on the Boruta algorithms, a total of 68 important variables were chosen as candidates for further investigation (Figure 4A). LASSO and SVM-RFE were utilized to further fit into LASSO regression distinguished 22 GM-related genes with an optimal lambda value of 0.0258 (Figures 4B–D). Analysis of the ROC curve revealed that the AUC of the LASSO model based on 22 genes was 0.97 in the training set and 0.95 in the test set (Figure 4E). In addition, based on the SVM-RFE model, the combination of 27 genes exhibited the highest precision for predicting AD, with a reliable AUC value in the training set (0.962) and the test set (0.964) (Figures 4F,G). Ultimately, the Venn plot indicated that PLXNA1, CES2, TMEM30A, PIK3C2A, LCOR, PDGFB, ATP13A4, CD164, and PHF1 were determined as the distinct GM-related genes after intersecting the results of the Lasso and SVM-RFE approaches (Figure 4H).

### Construction and validation of a diagnostic riskScore

**Figure 5:**
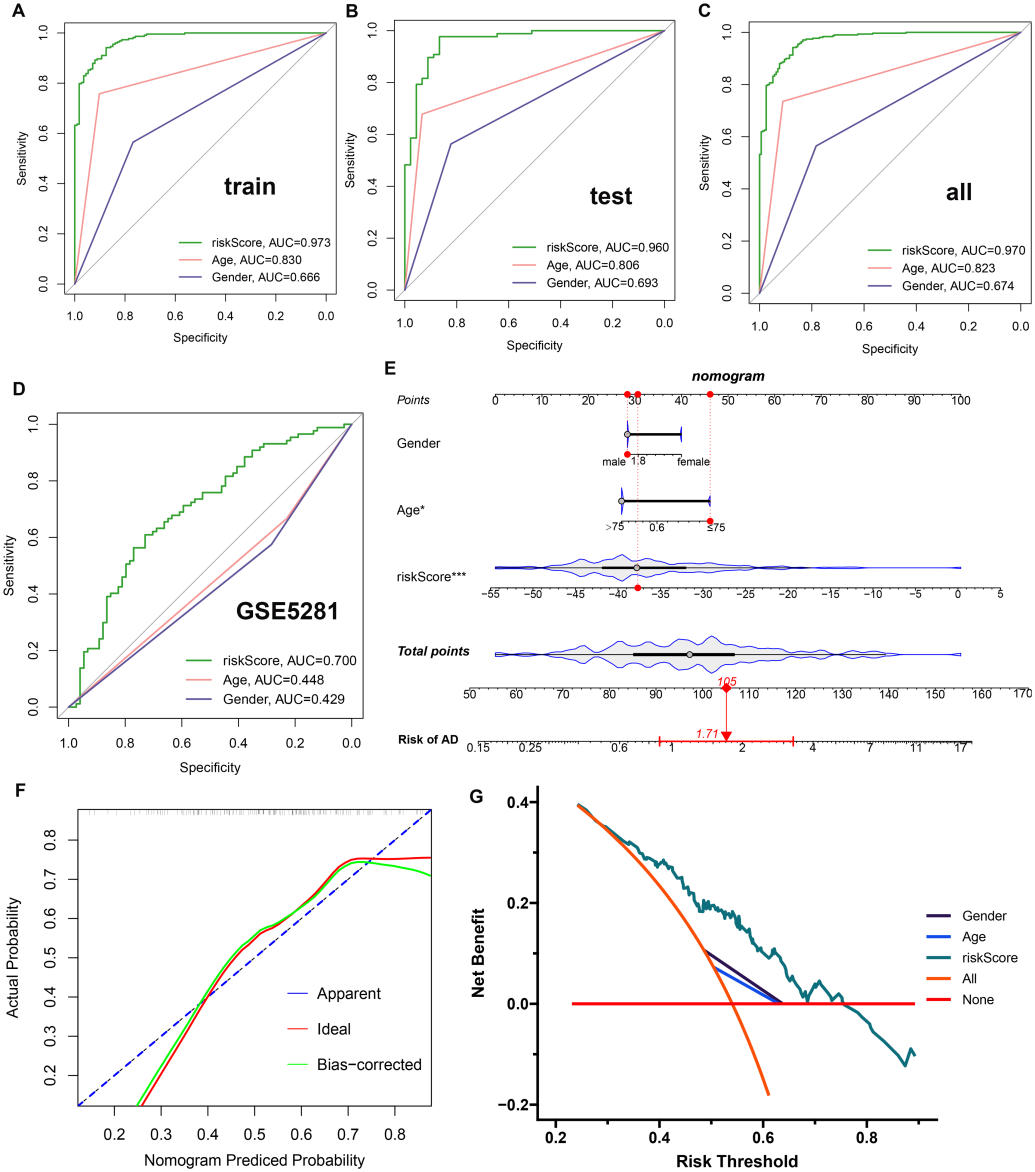
Construction and validation of a diagnostic riskScore. **(A–C)** The ROC curves illustrated the diagnostic efficacy of riskScore and typical clinical characteristics in the training cohort **(A)**, the test cohort **(B)**, and GSE33000 **(C)**. **(D)** The ROC curves illustrated the diagnostic efficacy of riskScore and typical clinical indicators in GSE5281. **(E)** Construction of a predictive nomogram based on risk scores and clinical characteristics in GSE5281. **(F)** Calibration curve for the appraisal of the reliability of a predictive nomogram. **(G)** DCA showed the clinical benefit of the nomogram.

A GM-related riskScore was established by using the following formula based on the corresponding LASSO model coefficients of the nine distinct genes: riskScore = (−7.424770887 × PLXNA1) + (−3.126593503 × CES2)+ (−2.200333626 × TMEM30A) + (−1.654918874 × PIK3C2A) + (0.403074115 × LCOR) + (0.418069564 × PDGFB) + (1.047735126 ATP13A4) + (3.209147766 × CD164) + (6.022106335 × PHF1). A ROC curve analysis was then used to compare the diagnostic accuracy of riskScore and clinical characteristics (age and gender) in predicting AD onset (Figures 5A–C). Compared to gender and age, riskScore showed higher AUC values in test, validation, and combined cohorts, indicating its higher potential for the diagnosis of AD. To further validate the efficacy of riskScore, we selected another dataset (GSE5281) for further analysis. The AUC values of the ROC curve for GSE5281 were 0.700 for riskScore, 0.448 for age, and 0.429 for gender, indicating that the riskScore maintained its better predictive potential (Figure 5D). Moreover, we constructed a nomogram consisting of clinical characteristics (age and gender) along with the riskScore for AD prediction, which also indicated that the riskScore was a superior predictor of AD progression compared to conventional clinical indicators (Figure 5E). We also used a calibration curve to confirm the stability of the nomogram (Figure 5F). In addition, the DCA curves of the nomogram demonstrated that AD patients may benefit from the nomogram-based clinical diagnosis (Figure 5G).

### Construction and molecular characteristic of a risk model

**Figure 6:**
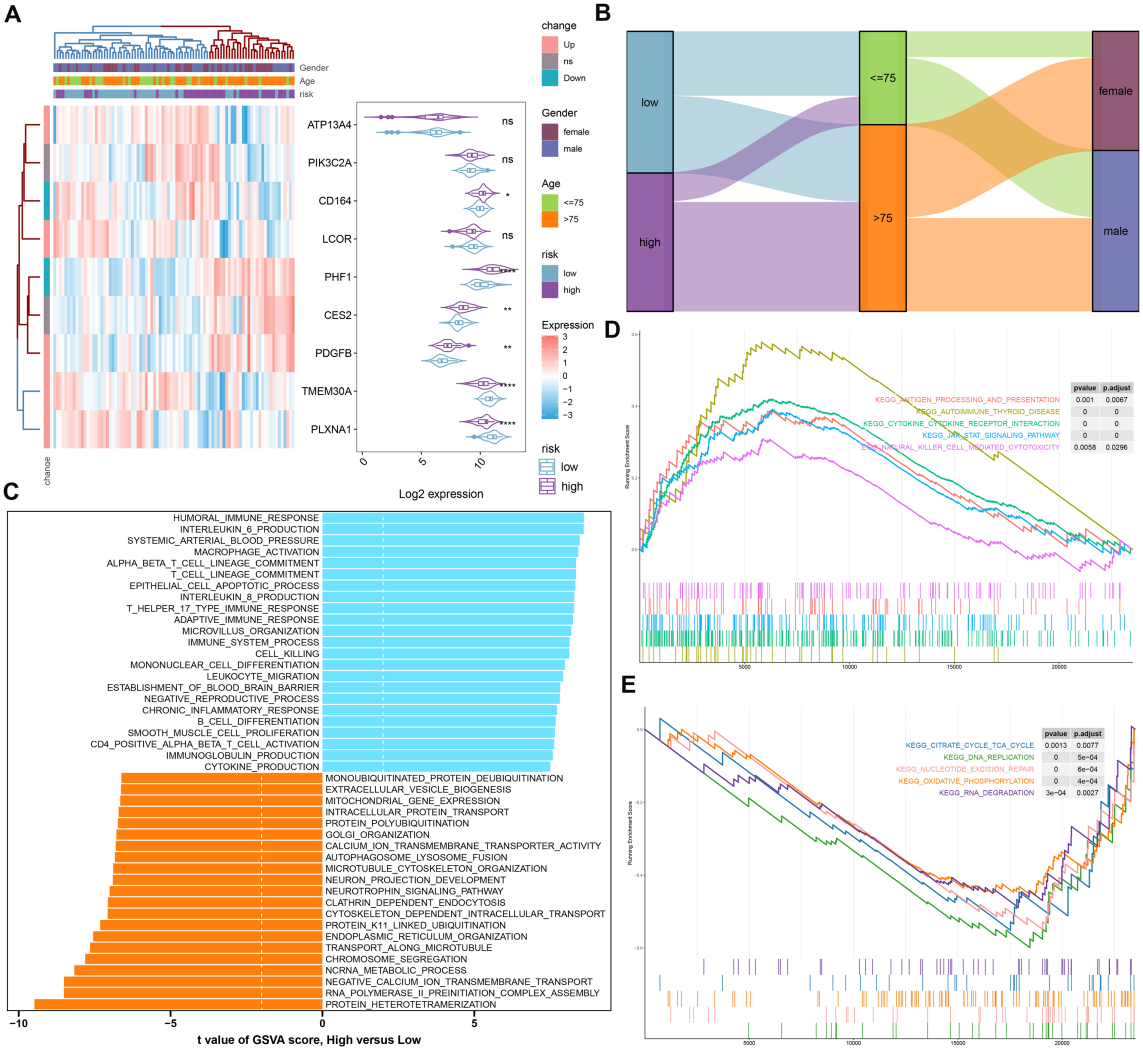
The construction and molecular characteristics of a risk model. **(A)** A heatmap displayed the expression profiles of 9 distinct genes associated with GM glutamine in low- and high-risk AD patients age, gender, and riskScore were exhibited as patient annotations. **(B)** The Sankey diagram showed the relationships among riskScore, age, and gender. **(C)** GSVA showed differences in biological function between high- and low-risk groups. **(D)** GSEA showed the top five upregulated pathways in the high-risk group. **(E)** GSEA showed the top five pathways down-regulated in the high-risk group.

To further elucidate the GM-related mechanisms in AD, we constructed a risk model based on the riskScore. The heat map and violin plot illustrated the varying expression patterns of nine GM-related genes in the low- and high-risk groups. CD164, PHF1, CES2, and PDGFB displayed higher levels of expression in the high-risk group, while TMEM30A and PLXNA1 showed significantly higher expression in the low-risk group. The expression of ATP13A4, PIK3C2A, and LCOR did not differ between the two groups (Figure 6A). The Sankey plot illustrated the distribution of age and gender in AD patients in two risk groups. We further concluded that in the high-risk group, the majority of patients were older than 75 years; however, there was no gender difference. Interestingly, in the low-risk group, AD patients were predominantly male, but there was no age discrepancy (Figure 6B).

We then annotated the enriched biological functions and selected significant enrichment pathways using GSVA and GSEA, respectively. In the high-risk group, immune response-related biological functions, including macrophage activation, immune response (T-helper 17 type immune response, adaptive immune response), chronic inflammatory response, cytokine production (IL-6, IL-8), leukocyte migration, CD4+ alpha-beta T cell activation, and immunoglobulin production, were highly enriched. The biological functions of the low-risk group were mainly involved in intracellular protein transport, organelle synthesis (endoplasmic reticulum organization, Golgi organization), and cytoskeleton-dependent intracellular transport (Figure 6C). According to GSEA, the low-risk group was primarily influenced by pathways related to the TCA cycle, DNA replication, nucleotide excision repair, oxidative phosphorylation, and RNA degradation (Figure 6E), while the high-risk group was particularly impacted by autoimmune thyroid disease, cytokine-cytokine receptor interactions, the JAK–STAT signaling pathway, and natural killer cell-mediated cytotoxicity (Figure 6D).

### Immune characteristics analysis

**Figure 7:**
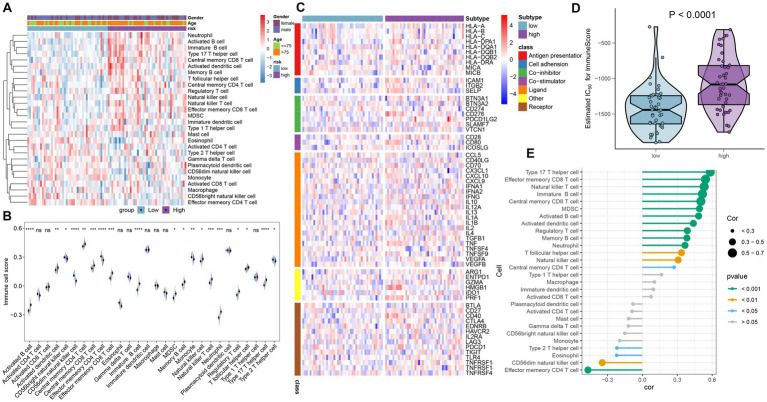
Immunological features of the high- and low-risk groups in AD patients. **(A)** The heat map showed the degree of infiltration of 24 immune cell subtypes in high- and low-risk groups based on the ssGSEA algorithm. **(B)** A split-half violin plot showed differences in 24 immune cell scores between high- and low-risk groups. **(C)** A heat map depicted the differences in immune-modulators between high- and low-risk groups. **(D)** A comparison of the immune score between high- and low-risk groups. **(E)** The interaction between the risk score and the 24 immune cell subtypes.

To demonstrate the landscape of infiltrating immune cells in the high- and low-risk groups, we first compared the differences in 28 immune cell subtypes and calculated the scores of 28 immune cells by the ssGSEA algorithm. A majority of immune cell subtypes, including activated B cells, activated dendritic cells, central memory CD4 T cells, central memory CD8 T cells, effector memory CD8 T cells, immature B cells, MDSC, memory B cells, natural killer cells, natural killer T cells, neutrophils, regulatory T cells, T follicular helper cells, and type 17 T helper cells, presented higher infiltration in the high-risk group compared with the other group. In contrast, the low-risk group got higher immune cell scores in CD56 bright natural killer cells, effector memory CD4 T cells, monocytes, and type 2 T helper cells (Figures 7A,B). Besides, the differences in immune modulators in the high- and low-risk groups were assessed to further elucidate the differences in immune characteristics of AD patients. The high-risk group showed significantly higher levels of immune genes associated with antigen presentation (HLA-DPA1, HLA-DQB1, HLA-DRA, and MICB), cell adhesion (ICAM1 and ITGB2), co-inhibitor (CD276, PDCD1LG2, and SLAMF7), co-stimulator (CD80), ligand (CD70, CXCL10, IL1B, and TNF), receptor (CD27 and CD40), and other immune-modulators (HMGB1 and PRF1) as depicted in Figure 7C. Immune scores from each risk group were compared, reflecting a qualitative assessment of immunological features. Patients in the high-risk group obtained higher immune scores than those in the low-risk group (Figure 7D). Furthermore, correlation analysis revealed that heightened riskScores were positively connected with the entirety of immune cell types and demonstrated higher levels of immune infiltration (Figure 7E).

### Validation of characteristic genes *in vitro*

**Figure 8:**
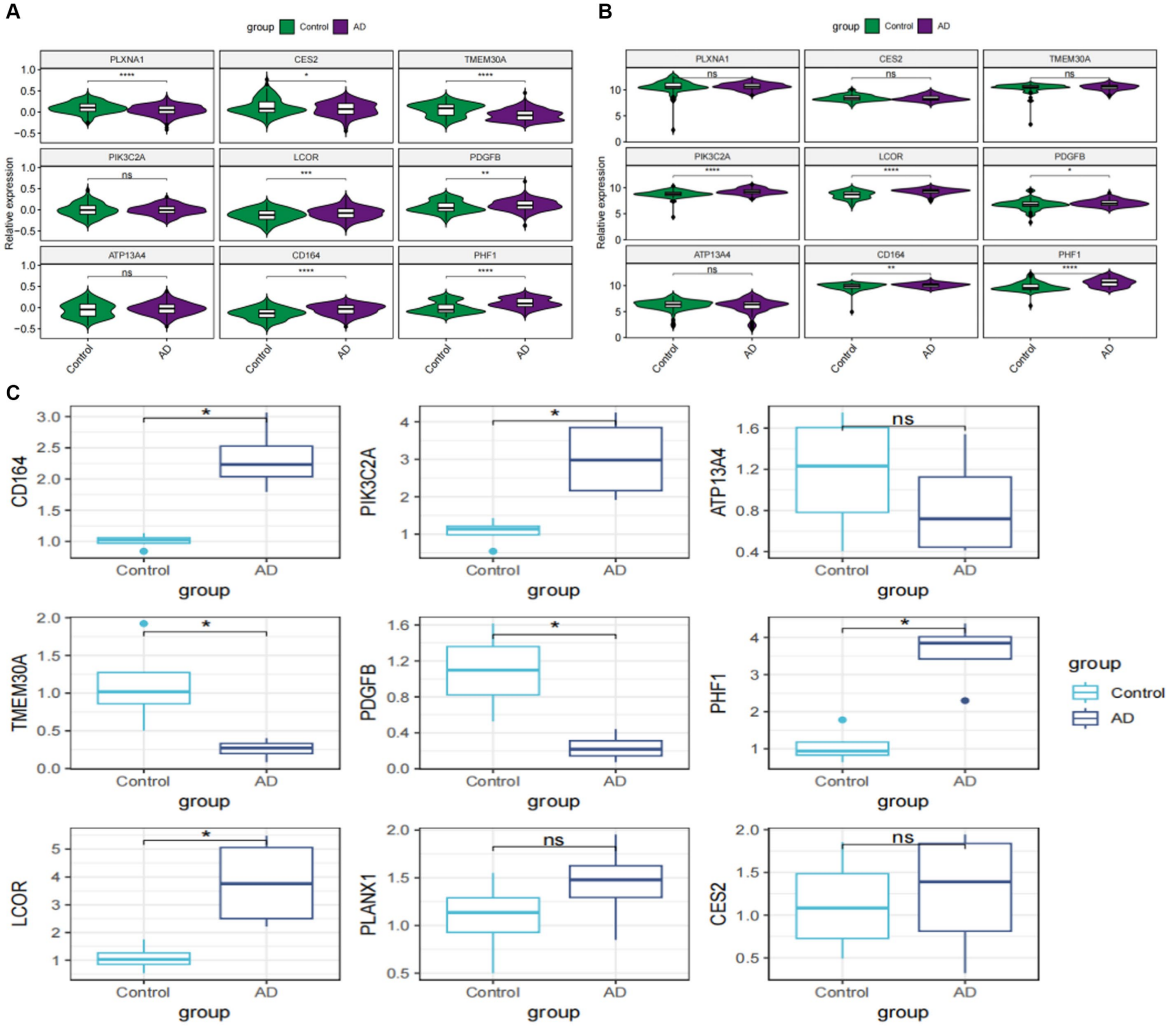
External validation of characteristic genes. **(A)** Violin plots showed the relative expression levels of characteristic genes in GSE33000. **(B)** Violin plots depicting the relative expression levels of characteristic genes in GSE5281. **(C)** Box plots reveal the expressional differences of characteristic genes based on RT-qPCR analysis *in vitro*. *p < 0.05, **p < 0.01, ***p < 0.001, ****p < 0.001.

To further investigate the expression landscape of nine characteristic genes related to GM, we analyzed their expression levels in the GSE33000 and GSE5281 datasets. LCOR, PDGFE, CD164, and PHF1 were substantially elevated in both GSE33000 and GSE5281 (Figures 8A,B). Additionally, we established an *in vitro* AD model of rat primary cortical neurons and analyzed the expression levels of these genes in both the model and control groups using the RT-qPCR technique. The majority of the nine characteristic genes showed different levels of expression. The expression levels of LCOR, CD164, and PHF1 were notably higher in AD cortical neurons, consistent with the results of the datasets (Figure 8C).

**Figure 9:**
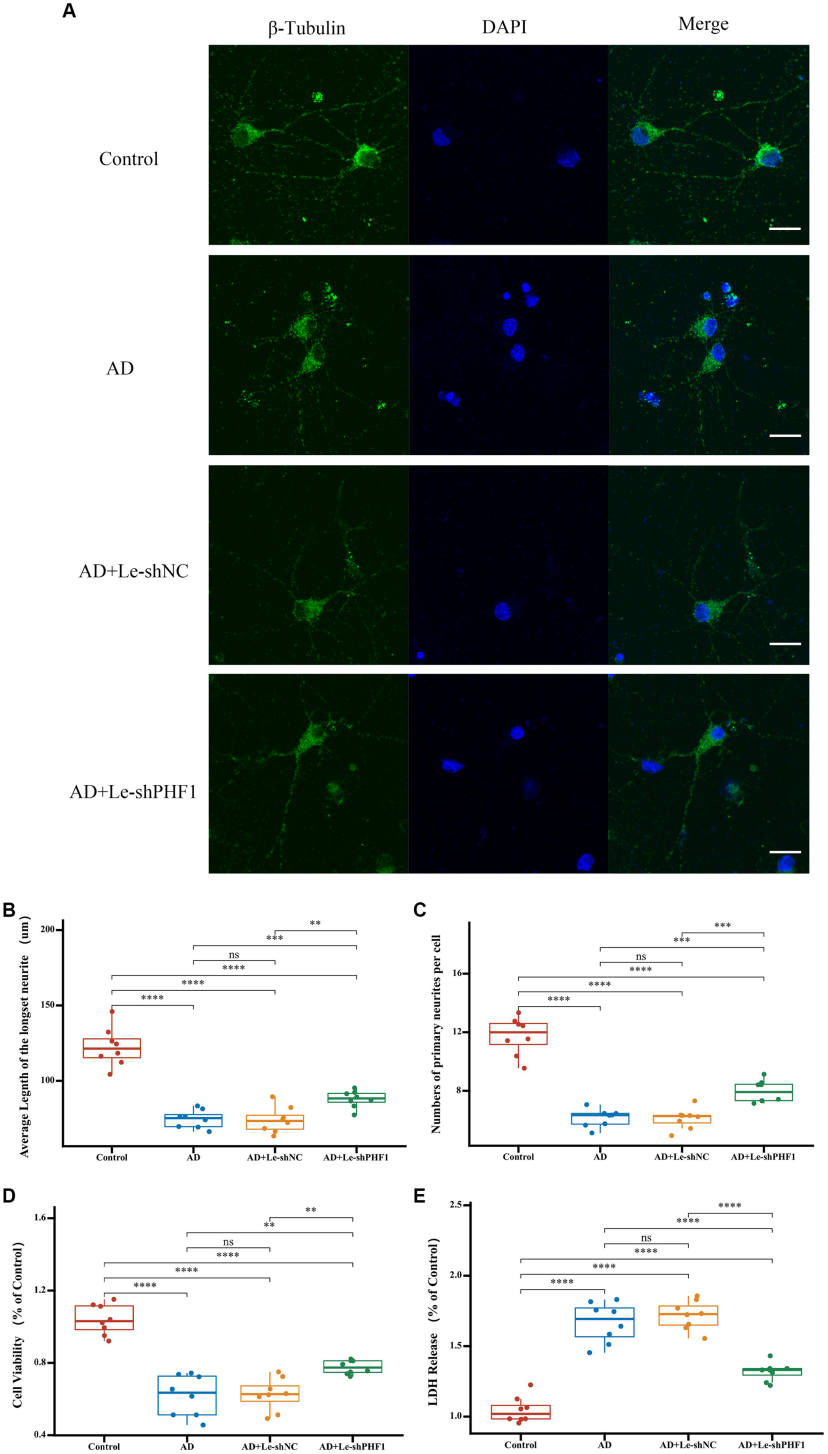
Detection of neurites and cell injury after PHF1 knockdown *in vitro*. **(A)** Immunofluorescence demonstrated the growth of neurites in each group. The scale bar = 20 μm. **(B)** Average Length of the longest neurite of neurons in each group. **(C)** Average number of primary neurites per cell in each group. **(D)** CCK-8 cell viability assay after PHF1 knockdown. **(E)** LDH release assay after PHF1 knockdown. *p < 0.05, **p < 0.01, ***p < 0.001, ****p < 0.001.

To further verify the role of PHF1 in AD, a PHF1 knockdown model was constructed *in vitro*. Compared to other feature genes identified in this study, it appeared that PHF1 played a crucial role within the risk scoring system due to obtaining the highest specific coefficient value. Consequently, an *in vitro* PHF1-knockdown model of AD was established. To verify the model’s performance, RT-qPCR was applied. In the Ad-shPHF1 group, the expression level of PHF1 was nearly four times lower than that in the AD+Ad-shNC group, affirming the model’s knockdown efficiency (Supplementary Figure S1). Immunofluorescence staining was employed to determine the mean length of the longest neurites and the average count of primary neurites (Figure 9A). Neurons in the AD model exhibited shorter neurite lengths and fewer primary neurites compared to the control group. However, PHF1 knockdown in AD neurons led to increased neurite length and higher numbers of primary neurites, suggesting a potential protective function against neurite loss during AD progression (Figures 9B,C). Furthermore, PHF1 knockdown in AD neurons indicated higher cell viability and reduced levels of LDH release compared to standard AD neurons (Figures 9D,E).

### Detection of glutamine and inflammatory factors after PHF1 knockdown *in vivo*

**Figure 10:**
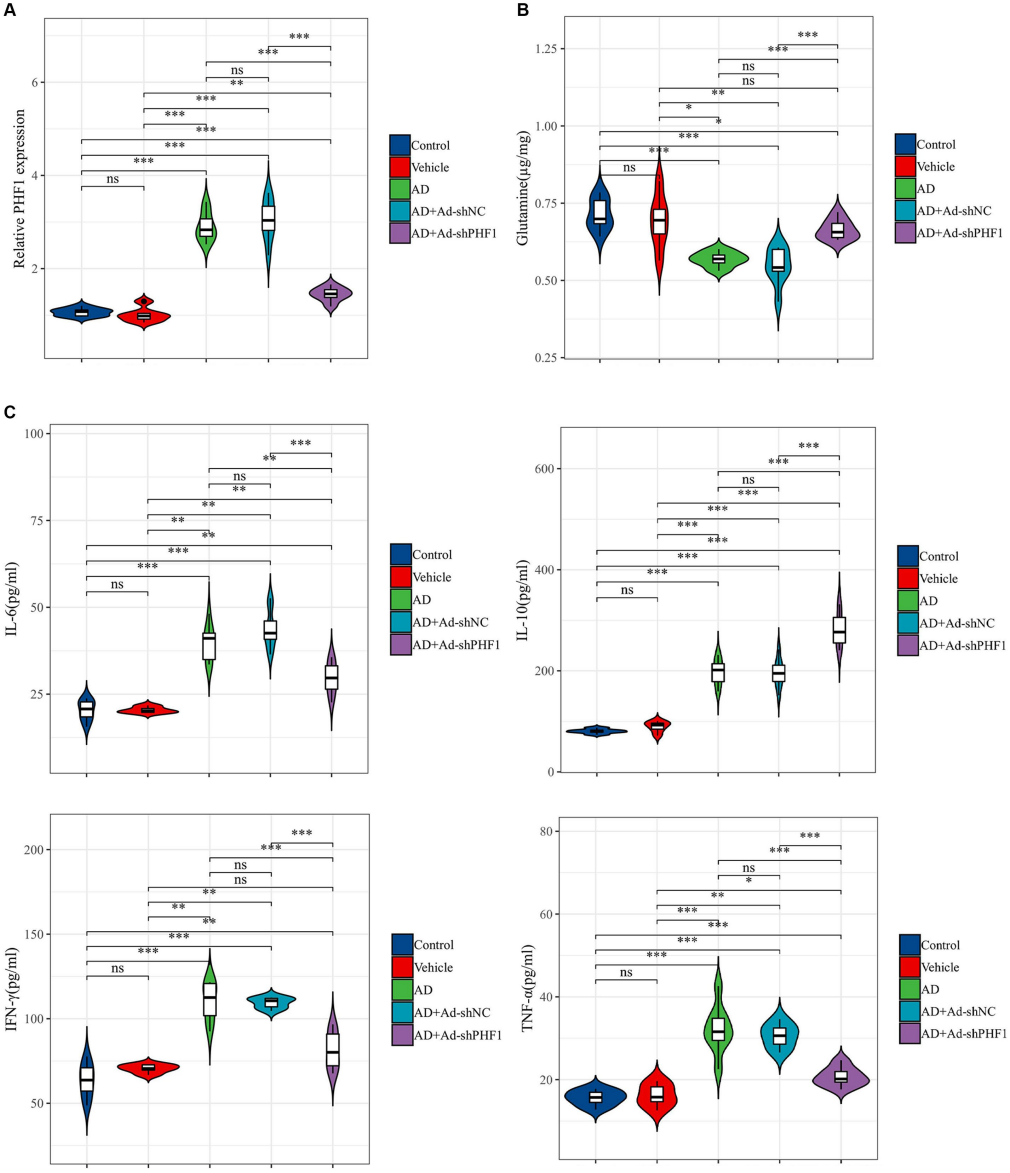
Detection of glutamine and inflammatory factors after PHF1 knockdown in AD rats. **(A)** The relative expression levels of PHF1 in the control, vehicle, AD, AD+Ad-shNC, and AD+Ad-shPHF1 group. **(B)** Glutamine levels in the brains of animals in each group. **(C)** Levels of inflammatory factors in the brains of animals in each group (*n* = 6 in each group). *p < 0.05, **p < 0.01, ***p < 0.001, ****p < 0.001.

An *in vivo* animal model of AD was established to investigate the role of PHF1 in glutamine metabolism and inflammation in AD. RT-qPCR was used to assess the performance of the knockdown model. The expression level of PHF1 in the AD+Ad-shPHF1 group was approximately three times lower than in the AD+Ad-shNC group, providing evidence of the efficiency of this knockdown model (Figure 10A). The level of glutamine in the brains of AD rats was lower compared to the levels in the control and vehicle groups. Intriguingly, AD rats with PHF1 knockdown exhibited higher glutamine levels compared to the AD group and AD+Ad-shNC group (Figure 10B). Moreover, elevated levels of pro-inflammatory factors (IL-6, IFN-γ, and TNF-α) were observed in the brains of AD subjects compared to the control group. Conversely, knockdown of PHF1 resulted in a reduction of these pro-inflammatory factors in the AD rat brain. Furthermore, the AD+Ad-shPHF1 group exhibited higher levels of the anti-inflammatory cytokine IL-10 compared to the AD group (Figure 10C).

## Discussion

Due to the diversity of AD, patients exhibit varying therapeutic efficacy and clinical outcomes, and there has been minimal progress in personalizing AD treatment. Therefore, identifying and understanding the heterogeneity of AD would aid in comprehending the pathology of this debilitating disease and developing more effective therapies. Recent studies have shown that the metabolism of certain amino acids in plasma, including branched-chain amino acids, glutamate, glutamine, and taurine, is linked to AD (Horgusluoglu et al., 2022). Specifically, glutamine is associated with AD, and impaired GM has been identified as a pathological process that occurs before the appearance of amyloid plaques in AD mouse models (Olabarria et al., 2011; Andersen et al., 2017). Previous studies have shown that could worsen cognitive impairment in patients with AD (Hashimoto et al., 2016; van der Lee et al., 2018). Other research has demonstrated that glutamine possesses neuroprotective properties and may serve as a promising target for the prevention of AD (Andersen et al., 2021, 2022; Sun et al., 2022, 2023). Therefore, it may be beneficial to explore potential modifications to glutamine in future research aimed at preventing this debilitating condition.

In this study, we performed single-cell analysis and bulk RNA-seq-based transcriptional analysis based on 118 metabolic regulators related to GM. We calculated GM scores using two methods, Ucell and ssGSEA and found that AD patients had higher GM scores than the control group in both analyses. This suggests that there is an abnormal glutamate-glutamine cycle during the pathological process of AD. Recent research has shown that the ratio of glutamate to glutamine is decreased in the blood plasma of patients with AD and amnestic moderate cognitive impairment (Wang et al., 2014). Interestingly, we found the level of glutamine was lower in the AD rat’s brain, suggesting that the level of GM may increase in AD. Moreover, previous studies have established a correlation between neurological diseases and increased glutamine activity, which leads to the neurotoxicity of glutamate (Li et al., 2017; Le et al., 2019). Based on both our own and previous research findings, we can hypothesize that an excessive production rate of glutamine may contribute to the development and progression of AD. However, more research is required to validate this assumption. Therefore, we developed a riskScore for AD based on nine characteristic genes related to GM and divided patients into high- and low-risk groups. Our results showed that the GM-related risk score had significantly higher diagnostic accuracy for AD compared to the classical clinical assessment criteria of age and gender. Among these nine GM-related genes, ATP13A4, PIK3C2A, CD164, PHF1, CES2, and PDGFB were found to be overexpressed in the high-risk group and are promising targets for AD prediction and treatment. ATP13A4 is a calcium transporter that operates within the endoplasmic reticulum (Vallipuram et al., 2010). Over-expression of ATP13A4 results in increased intracellular calcium levels (Biamino et al., 2016). Studies on families have revealed that ATP13A4 gene variants are associated with both schizophrenia and autism. PIK3C2A is a member of the phosphoinositide 3-kinase (PI3K) family and may be a risk factor for chronic stable angina and acute coronary syndrome (ACS) (Soliman et al., 2023). CD164 has been identified as a dependable marker for the specification of Hematopoietic Stem/Progenitor cells (Pellin et al., 2019) and as a potential therapeutic target for Sézary syndrome (Wysocka et al., 2014). Furthermore, PHF1, a crucial factor in epigenetic regulation and genome maintenance, has been discovered as a novel reader for histone H4R3 symmetric dimethylation. Its interaction with the PRMT5-WDR77-CRL4B complex leads to the induction of carcinogenesis (Liu et al., 2018). CES2 is an important enzyme involved in the metabolism of endogenous esters, ester-containing drugs, and environmental toxicants. A recent study has shown that CES2 can stimulate the expression of hepatocyte nuclear factor 4 (HNF4) protein, which helps to maintain the progenitor subtype of pancreatic ductal adenocarcinoma. This discovery makes CES2 a potential new target for the treatment of pancreatic cancer (Chen et al., 2022). PDGFB plays a crucial role in recruiting pericytes expressing platelet-derived growth factor receptor beta (PDGFRβ) to blood vessels. In two animal models, the lack of PDGFB in platelets resulted in increased hypoxia and epithelial-mesenchymal transition in initial tumors, elevated levels of circulating tumor cells, and increased spontaneous metastasis to the liver or lungs (Chen et al., 2022). Furthermore, the expression of LCOR, TMEM30A, and PLXNA1 was significantly higher in the low-risk group, indicating their potential involvement in slowing the progression of AD as neuroprotective factors. LCOR has been identified as a potential target to improve the efficiency of immune checkpoint blockade in triple-negative breast cancer. This is done by improving the tumor antigen processing/presentation machinery, independent of the IFN pathway. Additionally, inhibition of the MAPK signaling pathway through downregulating PLXNA1 has been found to reduce the proliferation, migration, invasion, and metastasis of esophageal squamous cell carcinoma (Wang et al., 2019). However, it should be noted that the relationship between the eight hallmark genes and the clinical AD progression has not been documented yet. It is noteworthy that TMEM30A is involved in various functions such as phospholipid transport, positive regulation of transport, and aminophospholipid flippase activity facilitation. According to a recent study, TMEM30A, which acts as a lipid flippase with P4-ATPase, has the potential to be a therapeutic target for AD by modulating vesicular trafficking through the asymmetric distribution of phospholipids (Kaneshiro et al., 2022). Additionally, our findings show that the high-risk group is primarily enriched in immune response-related functions and pathways, while the low-risk group is associated with intracellular substance synthesis and transport. The results of this study indicate that utilizing riskScore based on nine GM-related characteristic genes is a more effective method for predicting the progression of AD compared to using traditional clinical characteristics. This was demonstrated through the use of ROC curves, nomograms, calibration curves, and DCA. Overall, our findings suggest that the upregulation of six glutamine metabolism-related genes in the high-risk group may increase the risk of developing AD, whereas the upregulation of three other GM-related genes in the low-risk group exhibited a protective effect against AD.

The dysregulation of the immune system is a significant characteristic of AD. Numerous studies have identified pathological abnormalities in central and peripheral immune responses that fluctuate over time (Bettcher et al., 2021). However, despite the progress, the precise mechanisms involved in immunotherapy for treating AD remain poorly understood and highly controversial. Glutamine, acting as a primary source of energy for specific immune cells, may have a unique effect on immunological activation (Krzywkowski et al., 2001). Targeting the regulation of the gut microbiota (GM) has potential as an immunotherapy approach for various types of tumors (de Jonge et al., 2020; Udumula et al., 2021; Boyer et al., 2022). although its impact on the immune microenvironment of AD remains unclear and requires further investigation. Our study revealed that immune cells, such as plasmacytoid dendritic cells (pDCs), plasma cells, and T effector/memory helper T cells (LTB), showed significantly high GM scores at the single-cell level in AD. Moreover, plasma and pDC cells exhibited high interaction intensity and number with other immune cells, indicating their vital role in the GM-related immune microenvironment in AD. By investigating the ligand-receptor interactions, we found that immune cells with high GM scores exhibited greater intercellular communication through LGALS9-PTPRC/CD44/SORT1 and TNFRSF13B/TNFRSF13C-TNFRSF13 pathways. Conversely, MIF-CD74-CXCR4/CD74-CD44 pathways showed suppression. Intriguingly, we found that pDCs played a crucial role in the intercellular communication between immune cells in GM-associated processes. pDCs have been identified as a unique IFN-I-producing cell type, and their potential role in excessive cytokine production in autoimmune diseases has been proposed (Reizis, 2019). Throughout the progression of AD, the accumulation of danger-associated molecular patterns (DAMPs), such as beta-amyloid and hyperphosphorylated tau, continuously stimulate microglia, resulting in their prolonged activation. Chronic activation of microglia results in the secretion of an excess of pro-inflammatory cytokines, which, in turn, regulate further microglial responses (Lau et al., 2021). Targeting the cytokine context in the brain has been a long-standing objective in AD research. However, the modulation of cytokine signaling in AD to produce beneficial effects is still uncertain. Further research is needed to fully understand the complex relationship between GM, pDCs, and cytokines. Our results suggest that pDCs heavily involved in GM in AD. We employed single-cell methods to screen for immune cells and their associated signaling pathways that influence glutamine metabolism throughout the onset and progression of AD. Consequently, this discovery reveals novel and extremely promising targets for future prevention and treatment of AD.

Subsequently, our study examined the distribution of 28 immune cell subtypes and various immune modulators in the AD immunological microenvironment of high- and low-risk Gln score groups. We discovered that immune cell infiltration and immune scores were substantially higher in the high-risk group. Furthermore, co-stimulators, cell adhesion molecules, co-inhibitors, ligands, and receptor-associated immune genes were more prominently expressed in the high-risk group, indicating a more pronounced immune response. As a result of increased immunological scores, immune cell infiltration, and immune modulators, we identified a high-risk group with an immune phenotype that could greatly benefit from immunotherapy. In contrast, the low-risk group is characterized by a focus on intracellular substance synthesis and transport. This research highlights that the established risk model not only allows for the implementation of immunotherapy but also serves as a vital tool for personalized treatment and medicine in patients with AD.

The accumulation of phosphorylated tau is a prominent pathological characteristic of AD as it leads to synaptic impairment, neuronal dysfunction, as well as the formation of neurofibrillary tangles (Wang and Mandelkow, 2016). According to a recent study, it was demonstrated that PHF1 immunoreactive pTau primarily interacts with proteins within neurons in patients who have advanced AD (Drummond et al., 2020). In our study, PHF1 was identified as a crucial gene feature in the GM-related risk-scoring system. Suppression of PHF1 expression could alleviate neurite damage and neuronal injury in neurons affected by AD. *In vivo*, down-regulation of PHF1 in AD models reduces GM metabolism levels and modulates the immunoinflammatory response in the brain. Our findings suggest that PHF1 may play a crucial role in immunoinflammatory responses associated with GM in AD. Therefore, targeting PHF1 holds promise as a therapeutic approach for AD.

However, this study is limited by its retrospective nature and the small sample size obtained from public databases. Further validation of the results is required through multicenter prospective studies. Furthermore, to determine the clinical utility of AD patients with varying molecular subtypes and riskScores, it is recommended to consider larger sample sizes that provide more prognostic and therapeutic information.

## Conclusion

In this study, we performed a comprehensive analysis of the expression patterns of GM in AD using both single-cell and bulk transcriptomic approaches. Our findings revealed that GM displays heightened activity in AD patients, correlating with excessive immune activation. Moreover, we identified nine GM-associated characteristic genes and developed a diagnostic and risk prediction model for glutamine metabolism-related conditions. These insights into GM-associated heterogeneity in AD hold significant implications for devising individualized treatments for patients with this disease.

## Data availability statement

The original contributions presented in the study are included in the article/Supplementary material, further inquiries can be directed to the corresponding author.

## Ethics statement

The animal study was approved by Institutional Animal Care and Use Committee of Zhengzhou University. The study was conducted in accordance with the local legislation and institutional requirements.

## Author contributions

YG: Conceptualization, Data curation, Formal analysis, Software, Writing – review & editing. TZ: Data curation, Software, Writing – review & editing. XC: Writing – original draft. ZC: Investigation, Writing – review & editing.
