# Supplementary figures and images for: Development of a diagnostic and risk prediction model for Alzheimer’s disease through integration of single-cell and bulk transcriptomic analysis of glutamine metabolism
Source: Front Aging Neurosci. 2023 Nov 10;15:1275793. doi: 10.3389/fnagi.2023.1275793 (PMC10667556; doi:10.3389/fnagi.2023.1275793)

**
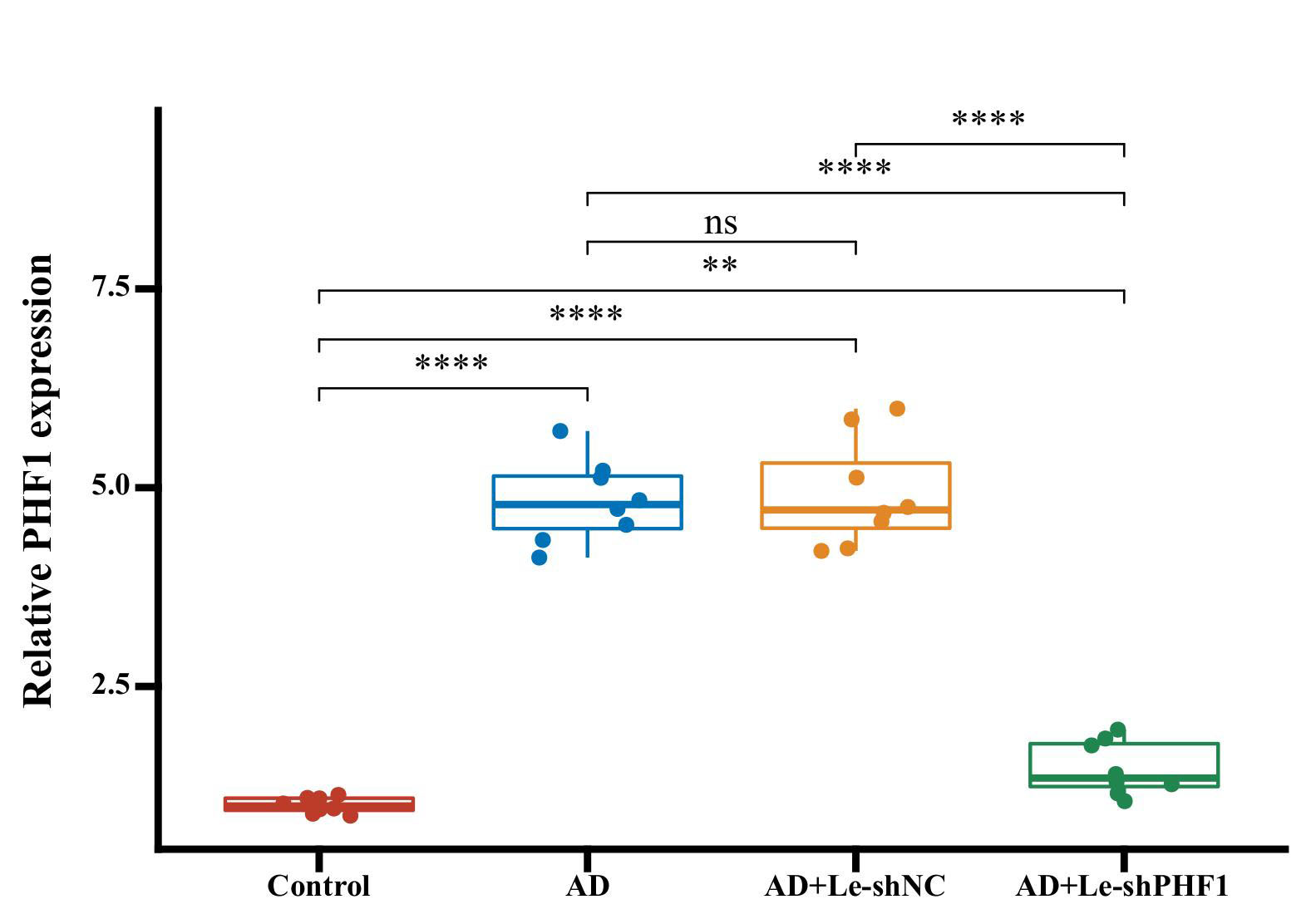
**

**Supplementary Figure S1** Validation of PHF1 knockdown efficiency in vitro

Supplement: Supplementary file 2 [file Data_Sheet_2.doc]
